# Concordance of blood- and tumor-based detection of *RAS* mutations to guide anti-EGFR therapy in metastatic colorectal cancer

**DOI:** 10.1093/annonc/mdx112

**Published:** 2017-03-20

**Authors:** J Grasselli, E Elez, G Caratù, J Matito, C Santos, T Macarulla, J Vidal, M Garcia, J M Viéitez, D Paéz, E Falcó, C Lopez Lopez, E Aranda, F Jones, V Sikri, P Nuciforo, R Fasani, J Tabernero, C Montagut, D Azuara, R Dienstmann, R Salazar, A Vivancos

**Affiliations:** 1 Department of Medical Oncology, Vall d’Hebron Institute of Oncology, Barcelona;; 2 Department of Medical Oncology, Catalan Institute of Oncology, Universitat de Barcelona, L’Hospitalet, Barcelona;; 3 Department of Medical Oncology, Vall d’Hebron University Hospital, Universitat Autònoma de Barcelona, Barcelona;; 4 Cancer Genomics Group, Vall d’Hebron Institute of Oncology, Barcelona;; 5 Department of Medical Oncology, Del Mar University Hospital, Barcelona;; 6 Department of Medical Oncology, Asturias University Hospital, Oviedo;; 7 Department of Medical Oncology, Santa Creu i Sant Pau University Hospital, Barcelona;; 8 Department of Medical Oncology, Son Llatzer University Hospital, Palma de Mallorca;; 9 Department of Medical Oncology, Marques de Valdecilla University Hospital, Santander;; 10 Department of Medical Oncology, Reina Sofía University Hospital, Córdoba, Spain;; 11 Sysmex Inostics, Mundelein, USA;; 12 Molecular Oncology Group, Vall d’Hebron Institute of Oncology, Barcelona;; 13 Traslational Research Laboratory, Catalan Institute of Oncology, L’Hospitalet, Barcelona;; 14 Oncology Data Science Group, Vall d’Hebron Institute of Oncology, Barcelona, Spain

**Keywords:** anti-EGFR therapy, circulating tumor DNA, metastatic colorectal cancer, RAS analysis

## Abstract

**Background:**

Circulating tumor DNA (ctDNA) is a potential source for tumor genome analysis. We explored the concordance between the mutational status of *RAS* in tumor tissue and ctDNA in metastatic colorectal cancer (mCRC) patients to establish eligibility for anti-epidermal growth factor receptor (EGFR) therapy.

**Patients and methods:**

A prospective-retrospective cohort study was carried out. Tumor tissue from 146 mCRC patients was tested for *RAS* status with standard of care (SoC) PCR techniques, and Digital PCR (BEAMing) was used both in plasma and tumor tissue.

**Results:**

ctDNA BEAMing *RAS* testing showed 89.7% agreement with SoC (Kappa index 0.80; 95% CI 0.71 − 0.90) and BEAMing in tissue showed 90.9% agreement with SoC (Kappa index 0.83; 95% CI 0.74 − 0.92). Fifteen cases (10.3%) showed discordant tissue-plasma results. ctDNA analysis identified nine cases of low frequency *RAS* mutations that were not detected in tissue, possibly due to technical sensitivity or heterogeneity. In six cases, *RAS* mutations were not detected in plasma, potentially explained by low tumor burden or ctDNA shedding. Prediction of treatment benefit in patients receiving anti-EGFR plus irinotecan in second- or third-line was equivalent if tested with SoC PCR and ctDNA. Forty-eight percent of the patients showed mutant allele fractions in plasma below 1%.

**Conclusions:**

Plasma *RAS* determination showed high overall agreement and captured a mCRC population responsive to anti-EGFR therapy with the same predictive level as SoC tissue testing. The feasibility and practicality of ctDNA analysis may translate into an alternative tool for anti-EGFR treatment selection.

## Introduction

In metastatic colorectal cancer (mCRC), treatment with anti-epidermal growth factor receptor (EGFR) monoclonal antibodies cetuximab or panitumumab has demonstrated efficacy in wild-type (WT) *RAS* mutations and it is now considered imperative this determination at the time of diagnosis [1, 2]. Formalin-fixed, paraffin-embedded (FFPE) tumor tissue with PCR analysis is currently used as standard of care (SoC) for *RAS* testing and is considered the gold standard [3].

Circulating-free DNA (cfDNA) is natural DNA present in the cell-free fraction of blood. Recent studies have suggested that genomic alterations in solid tumors may be characterized by studying the circulating tumor DNA (ctDNA) released from cancer cells into the plasma [4]. In mCRC, ctDNA is detected in almost all patients but the low abundance requires highly sensitive techniques to study mutations present at low frequencies. This approach represents a liquid non-invasive biopsy with a potential for determining *RAS* status. The main benefits are based on the safety and convenience associated with minimally invasive procedures, accessibility at any time point—that favor dynamic/evolutive evaluation—and is not affected by sample selection bias, although accuracy and concordance with tumor-based techniques has not been fully elucidated in patients from clinical practice [5–7].

Here, we carried out a concordance biomarker analysis of 146 mCRC patients using plasma and tissue-based *RAS* mutation testing with BEAMing and SoC techniques in both specimens. Discordant results were analyzed in-depth taking into consideration both technical and clinical conditions. We investigated the value of this determination in terms of progression-free survival (PFS) in patients who had received anti-EGFR as well as overall survival (OS) and mutant allele fraction (MAF) analysis.

## Materials and methods

### Study design

This prospective-retrospective study recruited patients candidate for therapy from three Spanish hospitals as well as from a phase II multicentric TTD ULTRA clinical trial (NCT01704703) for prospective biomarker investigation. It was approved by the ethics committees of each hospital and all patients provided written informed consent. Patients were required to have a diagnosis of mCRC with available tumor tissue for mutational analysis, have not received anti-EGFR agents before plasma collection, and have evidence of measurable disease according to Response Evaluation Criteria in Solid Tumors (RECIST) version 1.1 [8].

Plasma was obtained from 10 ml of blood and all patients had FFPE tissue (either primary tumor or metastasis) with >15% tumor area. Tumor tissue area was evaluated by the pathologist taking into consideration the amount of sample occupied by the tumor in a standardized procedure.

All samples were analyzed blinded to the study endpoints. Full description in supplementary methods, available at *Annals of Oncology* online.

### RAS mutational analysis

*RAS* status determination was carried out with available plasma and tumor tissue using BEAMing and Real-Time PCR as SoC technique. The DNA extracted from FFPE tissue sections was partitioned and used for both determinations (BEAMing and real-time PCR). The panel of *RAS* mutations evaluated with BEAMing was identical to that previously validated (supplementary Table S1, available at *Annals of Oncology* online) [2]. Each plasma and tumor sample was independently processed (using an 8-step workflow, supplementary Figure S1, available at *Annals of Oncology* online). In discordant cases the historical *RAS* reports were reviewed and further *RAS* determinations were carried out when metastases tissue was available, using SoC techniques (supplementary Table S2, available at *Annals of Oncology* online).

Depending on the specific assay, samples with a detectable mutation rate above 0.02%–0.04% were considered positive using BEAMing in ctDNA and 1% in tumor tissue. CtDNA testing was carried out with the commercially available CE-IVD BEAMing *RAS* plasma kit with the same thresholds for the specific mutations.

The sensitivity for Real-Time PCR as SoC analysis in tumor tissue is ∼1%–5%. Full description in supplementary methods and Table S3, available at *Annals of Oncology* online.

### Statistics

Full description in supplementary methods, available at *Annals of Oncology* online.

## Results

### Patient characteristics

A total of 157 mCRC patients were initially included, 11 of whom were excluded because of specific pre-analytical requirements or lack of tumor tissue availability (supplementary Figure S2, available at *Annals of Oncology* online).

Patient baseline characteristics, number and location of metastasis, and number and description of previous lines of therapy are summarized in supplementary Table S4, available at *Annals of Oncology* online.

Overall, 61 (42%) patients were naïve for therapy in the metastatic setting at the time of ctDNA collection, while the remaining 85 (58%) patients had received a range of treatments but all were anti-EGFR therapies naive. The median time from tumor tissue specimen to ctDNA collection was 1.2 months (range 0–34) in therapy-naive patients. The range in previously exposed patients was wide, with a median of 20.2 months (range 0.4–282). A group of 67 (46%) patients received anti-EGFR immediately after ctDNA collection mainly in second and third line (supplementary Table S4, available at *Annals of Oncology* online). Median PFS and median OS were described in supplementary Table S5, available at *Annals of Oncology* online.

### Correlation between RAS status in tissue and plasma

Using qPCR, we found tumor tissue samples positive for *KRAS* mutations in 44/146 samples (30%) and *NRAS* mutations in 10/146 (7%) (Table 1; supplementary Table S6, available at *Annals of Oncology* online). Using BEAMing in tissue samples, *KRAS* mutations were detected in 49/130 (38%) available samples and *NRAS* mutations in 11/130 (8%). For ctDNA analysis, 46/146 (31%) and 11/146 (8%) plasma samples harbored *KRAS* and *NRAS* mutations, respectively.

**Table 1:** Concordance between tumor-tissue and ctDNA analysis (*N *=* *146)

ctDNA analysis		Tumor-tissue analysis SoC			Sensitivity (%)	Specificity (%)	PPV (%)	NPV (%)
		*KRAS* mut	*NRAS* mut	WT				
BEAMing	*KRAS* mut	40	0	6	89	90	84	93
	*NRAS* mut	0	8	3				
	WT	4	2	83				
	Total	44	10	92				
		Tumor-tissue analysis BEAMing^a^			85	91	89	88
BEAMing	*KRAS* mut	42	0	4				
	*NRAS* mut	0	9	2				
	WT	7	2	64				
	Total	49	11	70				
Tumor-tissue analysis		Tumor-tissue analysis SoC			94	88	85	96
BEAMing	*KRAS* mut	42	0	7				
	*NRAS* mut	0	9	2				
	WT	2	1	67				
	Total	44	10	76				

a Tumor-tissue analysis with BEAMing was carried out in 130 samples.

WT, wild type; SoC, standard of care; ctDNA, circulating tumor DNA; PPV, positive predictive value; NPV, negative predictive value.

**Figure 1.**
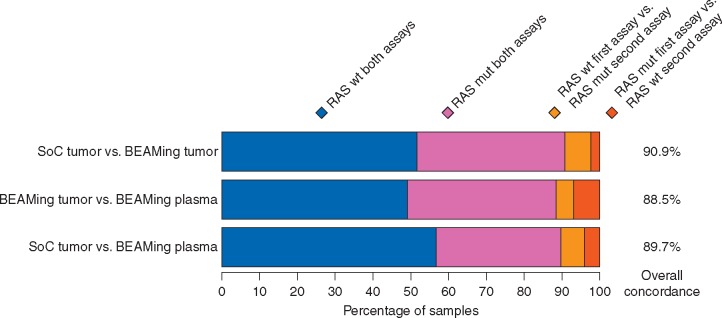
Concordance analysis. SoC tumor and BEAMing plasma analysis was carried out in 146 samples, BEAMing tumor was carried out in 130 samples. mut, mutation; SoC, standard of care.

Figure 1 shows concordance of *RAS* status between the three methods. ctDNA analysis showed a Cohen's Kappa estimate of 0.80 (95% CI 0.71–0.90) compared with tumor tissue evaluated by SoC reflecting almost perfect agreement according to Landis and Koch classification [9]. Results were similar for *RAS* status in plasma and tissue using BEAMing with a Kappa index of 0.79 (95% CI 0.69–0.89), and in tumor tissue using SoC and BEAMing a Kappa index of 0.83 (95% CI 0.74–0.92).

### Discordant samples description

In the population of samples with discordance between *RAS* status according to ctDNA BEAMing and tissue by SoC, two groups were identified, as detailed below (Table 2). To clarify these cases, the historical *RAS* testing was reviewed and additional *RAS* determinations were carried out by SoC in metastases whenever tissue was available (supplementary Table S2, available at *Annals of Oncology* online).

**Table 2:** Discordant samples

	ID	qPCR (SoC) tumor	BEAMing plasma	BEAMing tumor	**Additional (SoC) tumor**^c^	**Historical (SoC) tumor**^c^	Codon	MAF (BEAMing plasma)	adjMAF (BEAMing tumor)	Tissue source	Tissue tumor area (%)	Time tissue- plasma (month)	Previous chemo lines	**Previous treatment received**^d^	Anti-EGFR after plasma collection and best response	Possible explanation
Group A^a^	1	WT	MUT	MUT	MUT	WT	NRAS Q61	0.43	0.072	Primary	15	8	1	Capox adyuvant	FOLFIRI+ Panitumumab 1L (PR)	SoC sensitivity
	2	WT	MUT	MUT	MUT	WT	KRAS A146	0.0065	0.25	Primary	95	1	0		FOLFOX+ Cetuximab 1L (SD)	
	3	WT	MUT	MUT	NA	NA	KRAS A146	0.0061	0.29	Primary	50	7	0		No	
	4	WT	MUT	MUT	NA	MUT	KRAS G12	0.0006	0.058	Primary	20	108	2	5FU adyuvant, FOLFIRI 1L	No	
	5	WT	MUT	MUT	NA	MUT	NRAS Q61	0.0005	0.085	Primary	75	2	0		No	
	6	WT	MUT	WT	NA	WT	KRAS G12	0.0005		Metastasis	45	4	1	FOLFIRI+BVZ 1L	FOLFIRI+ Panitumumab 2L (PR)	Molecular heterogeneity
	7	WT	MUT	WT	NA	WT	KRAS G12	0.0008		Primary	70	3	0		No	
	8	WT	MUT	WT	WT	WT	KRAS Q61	0.0015		Primary	70	1	0		FOLFIRI+ Cetuximab 1L (PR)	
	9	WT	MUT	WT	NA	WT	NRAS Q61	0.0005		Primary	100	10	1	FOLFIRI 1L	FOLFIRI+ Panitumumab 2L (PD)	
Group B^b^	10	MUT	WT	MUT	MUT^e^	MUT	KRAS G12		0,27	Primary	50	1	0		No	Low tumor burden?
	11	MUT	WT	MUT	MUT	MUT	KRAS G12		0,14	Primary	40	33	2	FOLFOX 1L, FOLFIRI 2L	No	
	12	MUT	WT	MUT	NA	MUT	KRAS G12		0,12	Primary	95	3	0	In course FOLFOX 1L (PR)	No	Chemotherapy effect?
	13	NA	WT	MUT	NA	MUT	NRAS G13		NA	Primary	70	8	0	In course FOLFOX + BVZ 1L (SD)	No	
	14	NA	WT	MUT	NA	MUT	NRAS Q61		NA	Primary	70	4	0	In course FOLFOX 1L (PD)	No	
	15	MUT	WT	WT	NA	MUT^f^	KRAS Q61			Primary	60	3	0		No	Technical issues?

a Group A: mutations detected in plasma but not in tissue by SoC.

b Group B: mutation detected in tissue by SoC but not in plasma.

c Supplementary Table S2, available at *Annals of Oncology* online.

d In those patients with plasma extraction during chemotherapy immediate response after extraction is reported between brackets.

e Codon NRAS A59.

f Codon KRAS G13.

SoC, standard of care; MAF, mutant allele fraction; adjMAF, adjusted mutant allele fraction; Chemo, chemotherapy (adyuvant and/or metastatic setting); MUT, mutation; NA, not available; PR, partial response; SD, stable disease; PD, progression disease; 1L, frontline metastatic therapy; 2L, second line metastatic therapy; Capox, Capecitabine + oxaliplatin; 5FU, 5-fluorouracil; BVZ, Bevacizumab; FOLFIRI, 5FU + leucovorin + irinotecan; FOLFOX, 5FU + leucovorin + oxaliplatin.

Group A includes patients with evidence of mutations detected in plasma but not in tissue by SoC techniques. In the first five cases the SoC tissue technique failed to detect mutations that were detected in the same tumor sample by BEAMing (Table 2).

Interestingly, in cases 1 and 2, SoC analysis of additional metastatic samples showed the same mutations as those found in plasma supporting the concept that plasma can be used to capture tumor heterogeneity. Likewise, in cases 4 and 5 the historical reports showed identical mutated as plasma BEAMing but the new qPCR result was WT.

On the remaining four cases (ID 6–9) of this group the mutation detected by plasma BEAMing could not be identified by any other tumor sampling test. These cases appeared not to have specific clinicopathologic features or differential tissue sampling timing.

In group B, mutations were detected in tissue but not in plasma in six patients (Table 2). In this group, we also reviewed the CT scan carried out closest to the blood extraction to calculate tumor burden. Patient 10 had small hepatic lesions (<1.5 cm) and patient 11 had only three peritoneal lesions, both of which reflect low tumor burden. For three patients (ID 12–14), plasma extraction was carried out during the course of chemotherapy, which may have altered ctDNA detection. The immediate RECIST 1.1 response after plasma extraction was also reviewed. The last case (ID 15) had discordant results between tissue BEAMing and SoC evaluations even though the DNA for this analysis originated from the same tumoral tissue block. Again, these cases did not have any other particular clinic-pathologic features or differential time to tumor sampling.

### MAF analysis: distribution and median values

**Figure 2.**
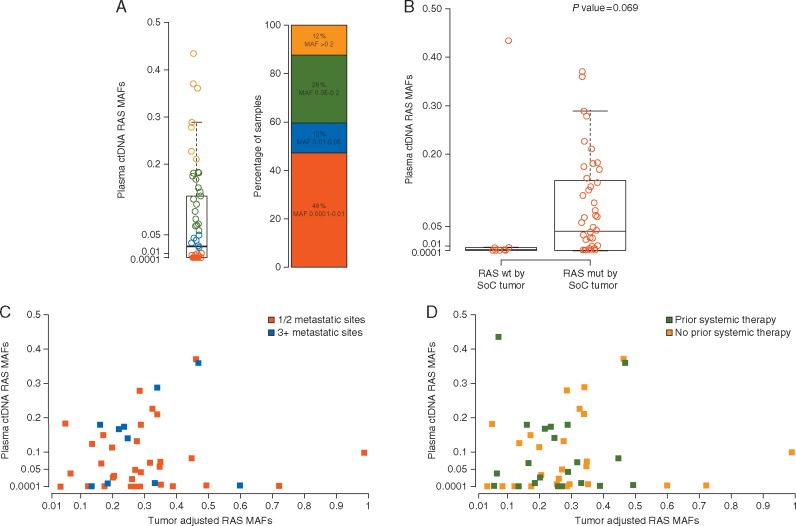
Mutant allele fraction analysis. (A) *RAS* mutant allele fractions in ctDNA BEAMing, a MAF of 0.01 corresponds to a percentage of mutant alleles of 1%. (B) Comparison of *RAS* mutant allele fractions in ctDNA and positivity for *RAS* mut tumor by SoC testing. (C) Correlation of *RAS* mutant allele fractions with BEAMing carried out in tumor (adjusted for purity) and ctDNA, according to prior systemic therapy exposure. Samples with *RAS* wild-type by SoC were excluded. (D) Correlation of *RAS* mutant allele fractions with BEAMing carried out in tumor (adjusted for purity) and ctDNA, according to number of metastatic sites. Samples with *RAS* wild-type by SoC were excluded. mut, mutation SoC, standard of care.

*RAS* MAFs had a median of 0.02 (range 0.0001–0.43) in plasma and were found in a wide distribution, 48% showed <1% (MAF <0.01) mutant alleles in their cfDNA (Figure 2A). *RAS*-adjusted MAFs had a median of 0.25 (range 0.03–0.99) in tumor tissue.

In the group of patients with concordant mutant samples in ctDNA and tissue by SoC (*N *=* *48), median MAF in plasma was 0.04 (range 0.0001–0.37) (Figure 2B). In the discordant cases (*n* = 9) median MAF was 0.0008 (range 0.0004–0.43) (*P *=* *0.069, Kruskal test).

In concordant samples by BEAMing tested in both tumor and plasma (*N *=* *48), median adjusted MAF was 0.26 (95% CI 0.04–0.99) in tumor and 0.14 (95% CI 0.05–0.99) (*P *=* *0.16, Kruskal test) in discordant samples (*N *=* *7). Overall, there was a tendency for lower MAFs both in tumor and plasma for the samples with discordant results.

The median MAF in ctDNA was also described according to prior chemotherapy exposure and number of metastatic sites. In the first case, median MAF was 0.07 (95% CI 0.002–0.16) and 0.04 (95% CI 0.006–0.15) in those with no prior therapy and those exposed, respectively (*P *=* *0.69, Kruskal test). In the second case, median MAF was 0.05 (95% CI 0.002–0.13) in those with one or two metastatic sites and 0.15 (95% CI 0.009–0.18) in those with three or more (*P *=* *0.24, Kruskal test).

### Correlation of MAF in concordant mutant samples in plasma and tissue

We carried out a *RAS-*adjusted MAF correlation analysis with BEAMing carried out in tumor and ctDNA in the same patient according to prior systemic therapy exposure or number of metastatic sites (Figure 2C and D). Mutational load showed very high heterogeneity and poor correlation, with a Pearson correlation coefficient in the overall population (*N *=* *43) of 0.10 (95% CI −0.21 to 0.39*, P *=* *0.54).

### RAS status and correlation with anti-EGFR treatment benefit

**Figure 3.**
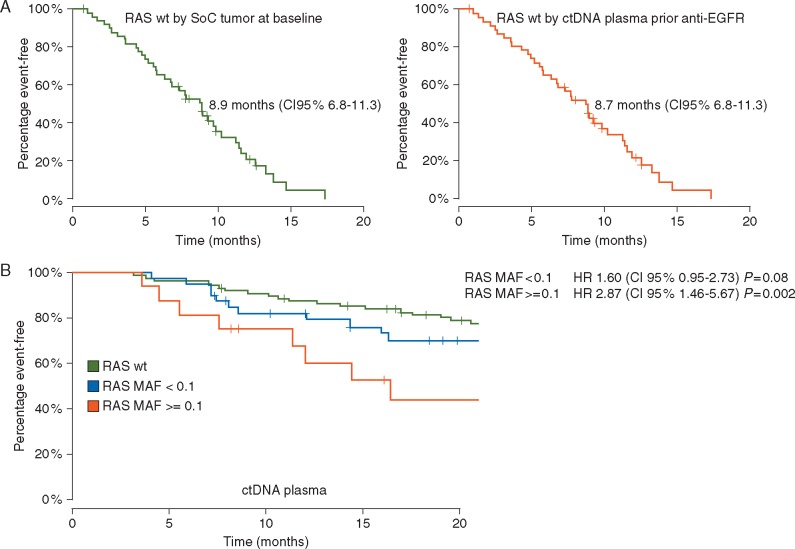
(A) Progression-free survival after anti-EGFR plus irinotecan-based therapy in the second or third-line setting in *RAS* wild-type metastatic colorectal cancer patients according to method of *RAS* mutation detection (SoC tumor tissue at baseline *N *=50 or ctDNA plasma before therapy *N *=47). (B) Survival in metastatic setting according to *RAS* mutant allele fraction by ctDNA plasma. MAF of 0.1 corresponds to a percentage of mutant alleles of 10%. SoC, standard of care.

The predictive value of *RAS* WT status from plasma and tumor determination was analyzed in the subset of patients who received anti-EGFR plus the irinotecan backbone in second- or third-line therapy (*N *=* *52). *RAS* WT patients detected by SoC (*N *=* *50) had a median PFS of 8.9 months (95% CI 6.8–11.3). *RAS* WT patients detected by ctDNA (*N *=* *47) showed a median PFS of 8.7 months (95% CI 6.8–11.3) (Figure 3A).

### Potential impact in OS

We describe outcomes for OS according to *RAS* MAF detection by ctDNA (Figure 3B). In the group of patients with *RAS* mutant samples with MAF < 0.1 by ctDNA (*N *=* *40), median OS was 27.8 months (95% CI 24.9–47.2), with an HR of 1.60 (95% CI 0.95–2.73; *P *=* *0.08) when compared with *RAS* WT population. In the group with MAF ≥0.1 (*n* = 16) median OS was 16.4 months (95% CI 11.4–not reached), and the HR for this group was 2.87 (95% CI 1.46–5.67, *P *=* *0.002) when compared with *RAS* WT population.

Relevant parameters were included in a multivariable Cox proportional hazards model on the entire cohort: mutation status and MAF in two ranges by ctDNA, tumor location and number of metastatic sites. *RAS* mutation with MAF ≥0.1 by ctDNA was shown to be a significant prognostic factor with a HR of 2.47 (95% CI 1.2–5.0, *P *=* *0.01) (supplementary Table S7, available at *Annals of Oncology* online).

## Discussion

This is the first clinical series showing the usefulness of detecting *RAS* point mutations by ctDNA in the largest cohort of patients published so far and carried out locally in a general hospital. Our data revealed a very high overall concordance, close to 90% compared with gold standard tumor tissue analysis techniques. This result is in accordance with previous reports, where *RAS* mutation detection in cfDNA has been directly compared with tumor tissue in CRC cohorts [4–6]. Siravegna et al. [7] focused on clonal evolution and resistance to EGFR blockade, also described excellent concordance in matched tissue and plasma samples using droplet digital PCR (*N *=* *100). Our results prove the feasibility for implementing this technique in the day-by-day care.

The detailed description of discordant samples reflected in Table 2 confirms the complexity of *RAS* genotyping in both tumor tissue and plasma samples. Translation of these new technologies to clinical practice reveal not only the technical limitations, but also bring to light conflicting data that provide information about the biological behavior of each tumor. Tumor tissue genotyping has inherent limitations the genomic profiles of primary tumors and metastases are not always concordant owing to the intrinsic molecular tumor heterogeneity [10, 11]. Likewise, several reports have shown differences ranging 3%–20% between different techniques to detect *RAS* mutations in tissue [12–14]. When analyzing tumor tissue by SoC and BEAMing analysis we detected a 9.1% rate of discordance, mostly justified by differences in sensitivity cut-off.

To account for spatial and temporal changes, the genomic profiles of CRC patients should be evaluated repeatedly during the course of therapy and liquid biopsies could play a role for determinations that are more representative of the specific molecular scenario of a patient at the time of anti-EGFR therapy selection [7, 15]. The possibility of *RAS* testing at the time of decision-making is one of the strongest points arguing in favor of this minimally invasive technique.

Furthermore, we consider several issues regarding *RAS* genotyping in plasma need to be highlighted. In our cohort, six patients had mutations in tissue that could not be detected in plasma. Lack of *RAS* mutations in plasma may be attributed to biological factors that impact ctDNA release and is an important matter that should be investigated. False negative results represent a major issue for *RAS* mutation testing on plasma because of the possible negative interaction of anti-EGFR agents with oxaliplatin-based regimens in *RAS* mutant patients.

Commonly used chemotherapeutic agents as well as targeted drugs can alter the molecular landscape in these tumors. It is widely acknowledged that acquired *KRAS* mutations are associated with secondary resistance to EGFR blockade [15, 16]. However, the effect on the molecular profile derived from other therapies such as anti-angiogenics or cytostatic agents before anti-EGFR administration is yet to be determined [17, 18]. Patients 6 and 9 (Table 2) may be such cases.

Tie et al. [19] reported changes in ctDNA for mCRC patients during the course of the chemotherapy, with significant reductions in ctDNA levels (median 5.7-fold) observed before cycle 2 in 41 of the 48 patients with concordant mutant samples in ctDNA and tissue by SoC. This could impact *RAS* status determination in patients exposed to therapy, we hypothesize that this could be the case for three patients in our cohort (ID 12–14 in Table 2), although we could not associate this with a homogeneous pattern of response. Taking this a step further, we detected a lower median MAF in the group of patients exposed to prior therapy.

If ultimately we move towards routine *RAS* determination in plasma in clinical practice, there will likely be subgroups of patients in whom we should continue to perform determinations in tissue for possible alterations in ctDNA release after a negative liquid biopsy.

Although the cohort size of patients with mutations (*N *=* *48) in our study is a somewhat limiting factor, we nonetheless could draw interesting conclusions from analyzing MAF, providing to our knowledge, the first published data in this field. When considering MAF distribution, a high proportion of patients showed mutant alleles in cfDNA between 0.0001 (0.01%) and 0.01 (1%). This highlights the importance of using an extremely sensitive technique when analyzing plasma samples and must be considered at the time of analysis to translate this into clinical practice. Interestingly, there is a tendency for lower MAFs both in tumor tissue and plasma for samples with discordant results, suggesting that sensitivity for mutation detection in tumor tissue is a real issue that needs to be addressed. We found no correlation of *RAS* MAF with BEAMing carried out in tumor and ctDNA, regardless of prior systemic therapies. The concept of a cut-off for plasma samples similar to that applied in tissue is complex and in our interpretation should not be equivalent.

Finally, in an exploratory analysis, and as an indirect way of confirming the possibility of selecting patients for anti-EGFR therapy with plasma, a PFS analysis was carried out in the most homogeneous group of our cohort, showing no relevant differences between detection methods. To our knowledge no other concordance studies have reported this, and this type of analysis is relevant to the implementation of liquid biopsies in clinical practice.

We can conclude that ctDNA analysis in plasma can detect RAS mutations to an equivalent level as SoC techniques in tissue, and thus detecting potential mCRC patients who could benefit from anti-EGFR therapies.

## Supplementary Material

mdx112_supp
