# Supplementary material for: Concordance of blood- and tumor-based detection of RAS mutations to guide anti-EGFR therapy in metastatic colorectal cancer
Source: Ann Oncol. 2017 Mar 20;28(6):1294–301. doi: 10.1093/annonc/mdx112 (PMC5834108; doi:10.1093/annonc/mdx112)
Supplement: mdx112_supp [file mdx112_supp.zip › Supplementary Table S7.docx]

**Supplementary Table S7: OS analysis of the entire cohort (multivariable analysis)**

|  | HR (CI95%) | *P* value |
| --- | --- | --- |
| Rectum vs. left | 0.97 (0.5-1.8) | 0.91 |
| Right vs. left | 1.69 (0.9-3.1) | 0.08 |
| Liver metastasis vs. no | 1.28 (0.7-2.3) | 0.43 |
| 3+ vs. 1-2 metastases | 1.67 (0.9-3.0) | 0.09 |
| *RAS* mut MAF <0.1^a^ vs. WT | 1.48 (0.9-2.6) | 0.16 |
| *RAS* mut MAF >=0.1 vs. WT | 2.47 (1.2-5.0) | **0.01** |

^a^MAF of 0.1 corresponds to a percentage of mutant alleles of 10%

Abbreviations:

vs, versus

mut, mutation
