# Supplementary material for: Concordance of blood- and tumor-based detection of RAS mutations to guide anti-EGFR therapy in metastatic colorectal cancer
Source: Ann Oncol. 2017 Mar 20;28(6):1294–301. doi: 10.1093/annonc/mdx112 (PMC5834108; doi:10.1093/annonc/mdx112)
Supplement: mdx112_supp [file mdx112_supp.zip › Supplementary methods .docx]

**Supplementary methods**

*Sample collection*

Non-trial patients plasma was obtained from 10 mL of blood collected in EDTA tubes and was isolated within 1 hour. Blood samples from patients in the TTD ULTRA clinical trial were collected in CellSave® preservative tubes and plasma was isolated within 48 hours.

A two-step centrifugation was performed with blood initially centrifuged for 10 minutes at 1,600 x *g* at room temperature. Supernatant was collected, avoiding the buffy coat, then centrifuged again to remove remaining cells, for 10 minutes at room temperature at 3000 x *g*. Plasma supernatant was transferred into a 1.5 mL tube and stored at -80ºC until use.

All patients had FFPE tissue (either primary tumor or metastasis) for mutational analysis which was evaluated by a dedicated pathologist to confirm the number of viable tumor cells. Ten 10-μm tissue sections with more than 15% tumor area were deparaffinized by xylene and ethanol extractions and subjected to proteinase K digestion overnight at 56ºC.

*DNA purification*

cfDNA was performed with the QIAamp Circulating Nucleic Acid Kit according to the manufacturer´s instructions, while extraction of FFPE samples (Ten 10-μm FFPE tissue sections each) was performed with the automated system Maxwell16 FFPE plus LEV DNA purification kit (Promega). DNA quality and concentration were measured with a NanoDrop 1000 spectrophotometer (Thermo Scientific, Waltham, MA).

*Mutation detection by BEAMing Technology in ctDNA and tumor*

cfDNA samples, obtained from 2 ml of plasma, were tested with the OncoBEAM RAS CRC Kit (catalog ZR150048) according to the manufacturer’s instructions (Sysmex Inostics). Flow-cytometry data acquired with the Cube 6i Flow Cytometer were analyzed using FCS Express^TM^ Software. Depending on the specific assay, samples with a detectable mutation rate above 0.02-0.04% were considered positive.

Tissue BEAMing was used to provide an independent gauge of *RAS* status in the tissue to be compared with results generated by the standard of care technique. FFPE tumor samples were shipped to the Sysmex Inostics service laboratory (Hamburg, Germany), where the tissue BEAMing was performed. Samples contained the minimum % of tumor content defined by the provider institution validated by the standard of care method. DNA extraction was performed using Qiagen QIAamp purification and quantification of the DNA using LINE-1 qRT-PCR for an abundant consensus region of the LINE-1 family and reference standard human genomic DNA. A basic minimum of 150ng of recovered DNA was required to perform BEAMing analyses; any samples not matching this basic criterion were deemed non-analyzable. Pre-amplification was performed with a first amplification of multiple loci in a multiplex PCR reaction, followed by a second pre-amplification with nested primers for individual amplicons. Emulsion PCR utilizes amplification on the surface of magnetic beads in oil-water emulsions subjected to thermal cycling. Hybridization to fluorescent labeled probes specific to the mutations of interest, followed by flow cytometry to quantify the resulting PCR product utilizing FCS Express Software to a ratio of mutant to wild-type alleles. Samples with a detectable mutation rate above 1% were considered positive.

*Real-Time PCR as standard of care analysis in tumor tissue*

Two-step PCR was performed to increase yield. In the first step, eight amplicons (four for *KRAS* and four for *NRAS*, **Supplementary Table S3 A**) were amplified for each sample. 50 ng of DNA was used for amplification with external primers, for 14 cycles of 30 seconds at 95ºC, 30 seconds at the corresponding annealing temperature (**Supplementary Table S3 A**), and 30 seconds at 72ºC, including a final extension step in a Mastercycler proS (Eppendorf). Amplified DNA was used as a template for nested PCR. The specific primers and probes for point mutations analyzed are detailed in **Supplementary Table S3 B.**

The real-time conventional assay (qPCR) was performed using a real-time PCR machine Light Cycler^®^ 480 (Roche Diagnostics, Applied Science). 1 μL of amplified genomic DNA was added to 2.5 μL TaqMan^®^ universal PCR Master Mix, 0.125 μL of 40X probes mix and 1.375 μL of H_2_0. The thermal cycling parameters were the same for all alleles tested: 95ºC for 10 minutes, 40 cycles at 92ºC for 15 seconds and annealing at 60ºC for 1 minute. A final step of allelic discrimination was necessary to differentiate between VIC and FAM signals. Light Cycler^®^ 480 Software v1.5 was used to determine the sample genotype by measuring the intensity distribution of the dyes after PCR. The sensitivity for this technique is approximately 1-5% (1). For 10 patients, data for tissue standard of care mutational status were taken from historical *RAS* determination by Therascreen Pyro^®^ and Cobas^®^ due to insufficient FFPE tissue that was prioritized for BEAMing tissue analysis**.** These 10 cases are included in the main qPCR standard of care analysis.

*Statistics*

For the sample size determination, a minimum concordance rate of 70% was assumed between standard of care tumor and ctDNA *RAS* testing (with an expected 40% prevalence of *RAS* mutations). On this basis, at least 140 samples were required to reach 90% power, with an alpha error of 0.05, to detect an expected 90% concordance rate.

Data are summarized by frequency for categorical variables and by median and range for continuous variables. PFS was defined as the time from anti-EGFR treatment start to disease progression or death. OS was defined as the time from mCRC diagnosis to death from any cause or the last follow-up visit. Response rate (RR) was assessed according to RECIST 1.1. Hazard ratios (HR) and 95% confidence intervals (CI) were calculated. Survival curves were estimated using the Kaplan-Meier method. Univariate and multivariable COX proportional hazards models were constructed for the endpoint of interest.

The diagnostic performance of ctDNA analysis was compared to tumor tissue analysis in distinguish­ing between mutated and non-mutated individuals. Concordance between the determinations was evaluated using the Cohen’s Kappa index.

MAF for a given case were calculated using the formula: Number of mutated alleles detected by a given probe/total number of alleles analyzed per genomic position. If measured in tumor tissue, MAFs were normalized to tumor purity. Adjusted MAF were calculated using the formula: MAF / tumor tissue area; and percentage of mutant alleles were calculated as MAF x 100. Pearson correlation coefficient was determined between MAF in tumor tissue (adjusted for purity) and in plasma using BEAMing was performed in 43 patients with *RAS* mutations in both samples. The non-parametric Kruskal-Wallis test was performed with the adjusted MAF for tumor and plasma using both BEAMing and PCR tumor determination, both of which included discordant cases.

Statistical analyses were performed using R 3.1.2 software, where *P* values of <0.05 were considered significant.

**References**

1. Azuara D, Ginesta MM, Gausachs M, et al. Nanofluidic digital PCR for KRAS mutation detection and quantification in gastrointestinal cancer. Clin Chem. 2012;58(9):1332-41.
