# Supplementary material for: Concordance of blood- and tumor-based detection of RAS mutations to guide anti-EGFR therapy in metastatic colorectal cancer
Source: Ann Oncol. 2017 Mar 20;28(6):1294–301. doi: 10.1093/annonc/mdx112 (PMC5834108; doi:10.1093/annonc/mdx112)
Supplement: mdx112_supp [file mdx112_supp.zip › Supplementary Table S1 .docx]

**Supplementary Table S1: *RAS* panel of mutations for BEAMing analysis**

| GEN | EXON | MUTATION |
| --- | --- | --- |
| KRAS | 2  2  3  3  4  4 | G12S/R/C/D/A/V  G13D  A59T  Q61L/H/R  K117N  A146T/V |
| NRAS | 2  2  3  3  4  4 | G12S/R/C/D/A/V  G13D/R/V  A59T  Q61K/L/R/H  K117N  A164T |
