# Supplementary material for: Concordance of blood- and tumor-based detection of RAS mutations to guide anti-EGFR therapy in metastatic colorectal cancer
Source: Ann Oncol. 2017 Mar 20;28(6):1294–301. doi: 10.1093/annonc/mdx112 (PMC5834108; doi:10.1093/annonc/mdx112)
Supplement: mdx112_supp [file mdx112_supp.zip › Supplementary Table S2 .docx]

**Supplementary Table S2: *RAS* determination in discordant cases**

|  | **ID** | **Additional determination** | | | **Historical determination** | | |
| --- | --- | --- | --- | --- | --- | --- | --- |
|  |  | **Tissue source** | **Technique** | **Result** | **Tissue source** | **Technique** | **Result** |
| Group A^a^ | 1 | Liver metastasis | CLART CMA KRAS-BRAF Kit, Therascreen NRAS and RAS extension Pyro Kit | MUT NRAS Q61 | Primary | CLART CMA KRAS-BRAF Kit, Therascreen NRAS and RAS extension Pyro Kit | WT |
|  | 2 | Peritoneal implant | Therascreen KRAS, NRAS and RAS extension Pyro Kit | MUT KRAS A146 | Primary | Therascreen KRAS, NRAS and RAS extension Pyro Kit | WT |
|  | 3 | NA |  |  | Primary | Therascreen KRAS, NRAS and RAS extension Pyro Kit | WT |
|  | 4 | NA |  |  | Primary | Cobas KRAS Mutation Test Kit | MUT KRAS codon 12/13 |
|  | 5 | NA |  |  | Primary | Therascreen KRAS, NRAS and RAS extension Pyro Kit | MUT NRAS Q61 |
|  | 6 | NA |  |  | Metastasis | qPCR Light Cycler | WT |
|  | 7 | NA |  |  | Primary | Therascreen KRAS, NRAS and RAS extension Pyro Kit | WT |
|  | 8 | Liver metastasis | CLART CMA KRAS-BRAF Kit, Therascreen NRAS and RAS extension Pyro Kit | WT | Primary | Therascreen KRAS mutation Kit qPCR | WT |
|  | 9 | NA |  |  | Primary | qPCR Light Cycler | WT |
| Group B^b^ | 10 | Liver metastasis | CLART CMA KRAS-BRAF Kit, Therascreen NRAS and RAS extension Pyro Kit | MUT NRAS A59 | Primary | Therascreen KRAS mutation Kit qPCR | MUT KRAS G12 |
|  | 11 | Peritoneal implant | CLART CMA KRAS-BRAF Kit, Therascreen NRAS and RAS extension Pyro Kit | MUT KRAS G12 | Primary | Therascreen KRAS mutation Kit qPCR | MUT KRAS G12 |
|  | 12 | NA |  |  | Primary | Cobas KRAS Mutation Test Kit | MUT KRAS codon 12/13 |
|  | 13 | NA |  |  | Primary | Therascreen KRAS, NRAS and RAS extension Pyro Kit | MUT NRAS G13 |
|  | 14 | NA |  |  | Primary | Therascreen KRAS, NRAS and RAS extension Pyro Kit | MUT NRAS Q61 |
|  | 15 | NA |  |  | Primary | Therascreen KRAS mutation Kit qPCR | MUT KRAS G13 |

^a^Group A: mutations detected in plasma but not in tissue by SoC

^b^Group B: mutation detected in tissue by SoC but not in plasma

Abbreviations:

SoC standard of care

NA, not available

MUT, mutation
