# Supplementary material for: Concordance of blood- and tumor-based detection of RAS mutations to guide anti-EGFR therapy in metastatic colorectal cancer
Source: Ann Oncol. 2017 Mar 20;28(6):1294–301. doi: 10.1093/annonc/mdx112 (PMC5834108; doi:10.1093/annonc/mdx112)
Supplement: mdx112_supp [file mdx112_supp.zip › Supplementary Table S3 .docx]

**Supplementary Table S3:**

**A** **Primers and conditions of PCR amplicons for qPCR analysis**

| **Gene** | **exon** | **amplicon** | **FW (5´→3´)** | **RV (5´→3´)** | **amplicon lenght (bp)** | **annealing Temperature (ºC)** |
| --- | --- | --- | --- | --- | --- | --- |
| *KRAS* | 2 | KRAS codons 12-13 | GGTGGAGTATTTGATAGTGTA | ATGGTCCTGCACCAGTAATATGCA | 243 | 52 |
| *KRAS* | 3 | KRAS codons 58-59-61 | CCAGACTGTGTTTCTCCCTTC | AAAGAAAGCCCTCCCCAGT | 152 | 61 |
| *KRAS* | 4 | KRAS codon 117 | TTTTTCTTTCCCAGAGAACAAAT | TCTTGTCTTTGCTGATGTTTCAA | 171 | 61 |
| *KRAS* | 4 | KRAS codon 146 | AGACACAAAACAGGCTCAGGA | GCCCTCTCAAGAGACAAAAACA | 219 | 61 |
| *NRAS* | 2 | NRAS codons 12-13 | GATGTGGCTCGCCAATTAAC | TGGTGGGATCATATTCATCTACA | 165 | 61 |
| *NRAS* | 3 | NRAS codons 58-59-61 | CACACCCCCAGGATTCTTAC | TGGCAAATACACAGAGGAAGC | 150 | 61 |
| *NRAS* | 4 | NRAS codon 117 | CCCGTTTTTAGGGAGCAGAT | TCTGGTCTTGGCTGAGGTTT | 168 | 61 |
| *NRAS* | 4 | NRAS codon 146 | TGGTGCTAGTGGGAAACAAG | GCAAACTCTTGCACAAATGC | 156 | 61 |

**B Panel of mutations, primers and probe sequences for qPCR analysis**

|  |  |  |  |  |  |  |  |  |  |  |
| --- | --- | --- | --- | --- | --- | --- | --- | --- | --- | --- |
| **GENE** | **MUT** | **EXON** | **CODON** | **CHANGE** | **PROT** | **FW (5´→3´)** | **RV (5´→3´)** | **Probe VIC** | **Probe FAM** | **Length (bp)** |
|  |  |  |  |  |  |  |  |  |  |  |
|  | 1 | 2 | 12 | 34G>T | G12C | GCCTGCTGAAAATGACTGAATATAAACT | GCTGTATCGTCAAGGCACTCTT | TTGGAGCTGGTGGCGTA | TTGGAGCTTGTGGCGTA | 78 |
|  | 2 | 2 | 12 | 35G>T | G12V | GCCTGCTGAAAATGACTGAATATAAACT | GCTGTATCGTCAAGGCACTCTT | TTGGAGCTGGTGGCGTA | TTGGAGCTGTTGGCGTA | 78 |
|  | 3 | 2 | 12 | 35G>A | G12D | GCCTGCTGAAAATGACTGAATATAAACT | GCTGTATCGTCAAGGCACTCTT | TTGGAGCTGGTGGCGTA | TTGGAGCTGATGGCGTA | 78 |
|  | 4 | 2 | 12 | 34G>C | G12R | GCCTGCTGAAAATGACTGAATATAAACT | GCTGTATCGTCAAGGCACTCTT | TTGGAGCTGGTGGCGTA | TTGGAGCTCGTGGCGTA | 78 |
|  | 5 | 2 | 12 | 34G>A | G12S | GCCTGCTGAAAATGACTGAATATAAACT | GCTGTATCGTCAAGGCACTCTT | TTGGAGCTGGTGGCGTA | TTGGAGCTAGTGGCGTA | 78 |
|  | 6 | 2 | 12 | 35G>C | G12A | GCCTGCTGAAAATGACTGAATATAAACT | GCTGTATCGTCAAGGCACTCTT | TTGGAGCTGGTGGCGTA | TTGGAGCTGCTGGCGTA | 78 |
|  | 7 | 2 | 13 | 38G>A | G13D | GCCTGCTGAAAATGACTGAATATAAACT | GAATTAGCTGTATCGTCAAGGCACT | CTTGCCTACGCCACCAG | CTTGCCTACGTCACCAG | 78 |
| **KRAS** | 8 | 3 | 58 | 173 C>T | T58I | GATGGAGAAACCTGTCTCTTGGAT | GGTCCCTCATTGCACTGTACTC | TTGACCTGCTGTGTCGAG | TTGACCTGCTATGTCGAG | 70 |
|  | 9 | 3 | 59 | 176 C>G | A59G | GATGGAGAAACCTGTCTCTTGGAT | CTGGTCCCTCATTGCACTGTA | TCCTCTTGACCTGCTGTGT | CTCCTCTTGACCTCCTGTGT | 72 |
|  | 10 | 3 | 59 | 175 G>A | A59T | GATGGAGAAACCTGTCTCTTGGAT | CCTCATTGCACTGTACTCCTCTT | CTCGACACAGCAGGTC | CTCGACACAACAGGTC | 66 |
|  | 11 | 3 | 61 | 183A>C | Q61H | GATGGAGAAACCTGTCTCTTGGAT | CCTCATGTACTGGTCCCTCATTG | CACTGTACTCCTCTTGACCT | ACTGTACTCCTCGTGACCT | 81 |
|  | 12 | 3 | 61 | 182 A>T | Q61L | GATGGAGAAACCTGTCTCTTGGAT | CATGTACTGGTCCCTCATTGCA | TGTACTCCTCTTGACCTGC | ACTCCTCTAGACCTGC | 78 |
|  | 13 | 3 | 61 | 182 A>G | Q61R | GATGGAGAAACCTGTCTCTTGGAT | CATGTACTGGTCCCTCATTGCA | TGTACTCCTCTTGACCTGC | ACTCCTCTCGACCTGC | 78 |
|  | 14 | 3 | 61 | 181 C>A | Q61K | GATGGAGAAACCTGTCTCTTGGAT | CATGTACTGGTCCCTCATTGCA | TGTACTCCTCTTGACCTGC | CTGTACTCCTCTTTACCTGC | 78 |
|  | 15 | 4 | 117 | 351 A>C | K117N (1) | TTAAGGACTCTGAAGATGTACCTATGGT | GAGCCTGTTTTGTGTCTACTGTTCT | CCTAGTAGGAAATAAATGTG | CCTAGTAGGAAATAACTGTG | 84 |
|  | 16 | 4 | 117 | 351 A>T | K117N (2) | TTAAGGACTCTGAAGATGTACCTATGGT | GAGCCTGTTTTGTGTCTACTGTTCT | CCTAGTAGGAAATAAATGTG | CCTAGTAGGAAATAATTGTG | 84 |
|  | 17 | 4 | 146 | 436G>A | A146T | GGCTCAGGACTTAGCAAGAAGTTAT | GCAGAAAACAGATCTGTATTTATTTCAGTGT | TCTTGTCTTTGCTGATGTT | TCTTGTCTTTGTTGATGTT | 93 |
|  | 18 | 4 | 146 | 437C>T | A146V | GGCTCAGGACTTAGCAAGAAGTTAT | GCAGAAAACAGATCTGTATTTATTTCAGTGT | CTGTCTTGTCTTTGCTGATG | CTGTCTTGTCTTTACTGATG | 93 |
|  | 19 | 4 | 146 | 436G>C | A146P | CAGGCTCAGGACTTAGCAAGAAG | GCAGAAAACAGATCTGTATTTATTTCAGTGT | AAACATCAGCAAAGAC | AAACATCACCAAAGAC | 93 |
|  | 20 | 2 | 12 | 34 G>T | G12C | CTGAGTACAAACTGGTGGTGGTT | GGATTGTCAGTGCGCTTTTCC | CAACACCACCTGCTCC | AACACCACATGCTCC | 60 |
|  | 21 | 2 | 12 | 35 G>T | G12V | GCTGGTGTGAAATGACTGAGTACAA | GGATTGTCAGTGCGCTTTTCC | TTGGAGCAGGTGGTGTT | TTGGAGCAGTTGGTGTT | 75 |
|  | 22 | 2 | 12 | 35 G>A | G12D | GCTGGTGTGAAATGACTGAGTACAA | GGATTGTCAGTGCGCTTTTCC | TTGGAGCAGGTGGTGTT | TTGGAGCAGATGGTGTT | 75 |
|  | 23 | 2 | 12 | 35 G>C | G12A | GCTGGTGTGAAATGACTGAGTACAA | GGATTGTCAGTGCGCTTTTCC | TTGGAGCAGGTGGTGTT | TGGAGCAGCTGGTGTT | 75 |
| **NRAS** | 24 | 2 | 13 | 37 G>C | G13R | CTGAGTACAAACTGGTGGTGGTT | CTGGATTGTCAGTGCGCTTTT | CCCAACACCACCTGCT | CCAACACGACCTGCT | 62 |
|  | 25 | 3 | 59 | 175 G>A | A59T | GGTGAAACCTGTTTGTTGGACATAC | GTCTCTCATGGCACTGTACTCTT | CTTGTCCAGCTGTATCC | CTTGTCCAGTTGTATCC | 66 |
|  | 26 | 3 | 61 | 181C>A | Q61K | GGTGAAACCTGTTTGTTGGACATAC | CCTGTCCTCATGTATTGGTCTCTCA | CTGTACTCTTCTTGTCCAGC | CTGTACTCTTCTTTTCCAGC | 83 |
|  | 27 | 3 | 61 | 182A>G | Q61R | GGTGAAACCTGTTTGTTGGACATAC | TGGTCTCTCATGGCACTGTACT | ACAGCTGGACAAGAAG | ACAGCTGGACGAGAAG | 68 |
|  | 28 | 3 | 61 | 183 A>T | Q61H | GGTGAAACCTGTTTGTTGGACATAC | CCTGTCCTCATGTATTGGTCTCTCA | CACTGTACTCTTCTTGTCCAG | ACTGTACTCTTCATGTCCAG | 83 |
|  | 29 | 3 | 61 | 182 A>T | Q61L | GGTGAAACCTGTTTGTTGGACATAC | GTATTGGTCTCTCATGGCACTGTAC | CAGCTGGACAAGAAGA | ATACAGCTGGACTAGAAGA | 72 |
|  | 30 | 4 | 117 | 351 A>C | K117N | AAGACTCGGATGATGTACCTATGGT | GGCTTGTTTTGTATCAACTGTCCTT | AGTGGGAAACAAGTGTGATT | TAGTGGGAAACAACTGTGATT | 80 |
|  | 31 | 4 | 146 | 436G>A | A146T | CCAAGAGTTACGGGATTCCATTCAT | AAATGCTGAAAGCTGTACCATACCT | AACCTCAGCCAAGACC | AAACCTCAACCAAGACC | 73 |
|  | 32 | 4 | 146 | 437 C>T | A146V | CCAAGAGTTACGGGATTCCATTCAT | AAATGCTGAAAGCTGTACCATACCT | TCTGGTCTTGGCTGAGGT | TCTGGTCTTGACTGAGGT | 73 |
|  | 33 | 4 | 146 | 436 G>C | A146P | CCAAGAGTTACGGGATTCCATTCAT | AAATGCTGAAAGCTGTACCATACCT | AACCTCAGCCAAGACC | ACCTCACCCAAGACC | 73 |
