# Supplementary material for: Concordance of blood- and tumor-based detection of RAS mutations to guide anti-EGFR therapy in metastatic colorectal cancer
Source: Ann Oncol. 2017 Mar 20;28(6):1294–301. doi: 10.1093/annonc/mdx112 (PMC5834108; doi:10.1093/annonc/mdx112)
Supplement: mdx112_supp [file mdx112_supp.zip › Supplementary Table S4.docx]

**Supplementary Table S4: Baseline demographic and clinical characteristics**

|  |  | **Number** | **Percentage** |
| --- | --- | --- | --- |
| Age in years | Median (range) | 65 (30-86) | - |
| Gender | Male | 106 | 73% |
|  | Female | 40 | 27% |
| Stage at diagnosis | Early | 46 | 32% |
|  | Advanced | 100 | 68% |
| Primary site | Right | 45 | 31% |
|  | Left | 55 | 38% |
|  | Rectum | 46 | 31% |
| Tumor tissue for *RAS* testing | Primary | 121 | 85% |
|  | Metastatic | 22 | 15% |
| Metastatic site at ctDNA collection | Liver | 101 | 69% |
|  | Lung | 59 | 40% |
|  | Node | 45 | 31% |
|  | Peritoneal | 31 | 21% |
|  | Other | 27 | 18% |
| Number metastatic sites at ctDNA collection | 1 | 66 | 45% |
|  | 2 | 53 | 36% |
|  | 3+ | 27 | 19% |
| Therapy prior to ctDNA collection | None | 53 | 36% |
|  | 5-FU | 93 | 64% |
|  | Oxaliplatin | 72 | 49% |
|  | Irinotecan | 60 | 41% |
|  | Anti-angiogenic | 42 | 28% |
|  | Anti-EGFR | 0 | 0% |
| Number of therapies for metastatic CRC prior to ctDNA collection | 0 | 61 | 42% |
|  | 1 | 45 | 31% |
|  | 2 | 34 | 23% |
|  | 3+ | 6 | 4% |
| Anti-EGFR therapy immediately after ctDNA collection | Yes | 67 | 46% |
|  | No | 79 | 54% |
| Anti-EGFR regimen | Irinotecan/anti-EGFR mAb | 64 | 95% |
|  | Oxaliplatin/anti-EGFR mAb | 1 | 2% |
|  | anti-EGFR alone | 2 | 3% |
| Anti-EGFR therapy line | First-line | 13 | 19% |
|  | Second-line | 20 | 30% |
|  | Third-line or later | 34 | 51% |
| Anti-EGFR therapy response | Complete | 2 | 3% |
|  | Partial | 36 | 53% |
|  | Stable | 24 | 35% |
|  | Progression | 6 | 9% |

Abbreviations:

mAb, monoclonal antibody
