# Supplementary material for: Concordance of blood- and tumor-based detection of RAS mutations to guide anti-EGFR therapy in metastatic colorectal cancer
Source: Ann Oncol. 2017 Mar 20;28(6):1294–301. doi: 10.1093/annonc/mdx112 (PMC5834108; doi:10.1093/annonc/mdx112)
Supplement: mdx112_supp [file mdx112_supp.zip › Supplementary Table S5.docx]

**Supplementary Table S5: Progression-free survival and overall survival description**

|  |  | **median (95%CI)** |
| --- | --- | --- |
| Median PFS with anti-EGFR therapy | All patients (*N*=67, 52 events) | 9.0 m (6.8-11.9) |
|  | First-line (*N*=13, 8 events) | 17.7 m (13.8-not reached) |
|  | Second-line (*N*=20, 14 events) | 7.3 m (4.5-not reached) |
|  | Third-line or later (*N*=34, 30 events) | 7.7 m (5.7-11.3) |
|  | Complete or partial response (*N*=38, 28 events) | 11.6 m (9.9-14.7) |
|  | Stable disease (*N*=23, 18 events) | 5.4 m (4.3-7.7) |
|  | Progression disease (*N*=6, 6 events) | 1.4 m (1.2-not reached) |
| Median OS in metastatic setting | All patients (*N*=144, 71 events) | 35.9 m (29.7-47.2) |
|  |  |  |

Abbreviations:

m, month
