# Supplementary material for: Concordance of blood- and tumor-based detection of RAS mutations to guide anti-EGFR therapy in metastatic colorectal cancer
Source: Ann Oncol. 2017 Mar 20;28(6):1294–301. doi: 10.1093/annonc/mdx112 (PMC5834108; doi:10.1093/annonc/mdx112)
Supplement: mdx112_supp [file mdx112_supp.zip › Supplementary Table S6.docx]

**Supplementary Table S6: Mutation analysis**

| **Mutation analysis from qPCR tumor tissue (*N*= 146)** | | | | |
| --- | --- | --- | --- | --- |
|  |  |  | **Number** | **Percentage** |
| ***RAS*** |  |  |  |  |
| Mutant |  |  | 54 | 37 |
| Wild type |  |  | 92 | 63 |
| **Mutation Type** | |  |  |  |
| KRAS G12D | |  | 12 | 8 |
| KRAS G13D | |  | 9 | 6 |
| KRAS G12V | |  | 7 | 5 |
| KRAS G12C | |  | 4 | 2 |
| KRAS A146T | |  | 3 | 2 |
| KRAS Q61H | |  | 2 | 1 |
| KRAS Q61L | |  | 2 | 1 |
| KRAS G12A | |  | 1 | 1 |
| KRAS G12C/A146T | |  | 1 | 1 |
| KRAS G12D/G12V | |  | 1 | 1 |
| KRAS G12V/A146T | |  | 1 | 1 |
| KRAS K117N | |  | 1 | 1 |
| NRAS Q61K | |  | 3 | 2 |
| NRAS G12D | |  | 2 | 1 |
| NRAS Q61R | |  | 2 | 1 |
| NRAS G12C | |  | 1 | 1 |
| NRAS G13R | |  | 1 | 1 |
| NRAS Q61L | |  | 1 | 1 |
| **Mutation analysis from BEAMing tumor tissue (*N*= 130)** | | | | |
|  |  |  | **Number** | **Percentage** |
| ***RAS*** |  |  |  |  |
| Mutant |  |  | 60 | 46 |
| Wild type |  |  | 70 | 54 |
| **Mutation Type** | |  |  |  |
| KRAS G12D | |  | 13 | 10 |
| KRAS G12V | |  | 9 | 7 |
| KRAS G13D | |  | 9 | 7 |
| KRAS A146T | |  | 7 | 5 |
| KRAS G12C | |  | 4 | 3 |
| KRAS G12A | |  | 2 | 1 |
| KRAS G12C/A146T | |  | 1 | 1 |
| KRAS G12D/G12V | |  | 1 | 1 |
| KRAS K117N | |  | 1 | 1 |
| KRAS Q61H | |  | 1 | 1 |
| KRAS Q61L | |  | 1 | 1 |
| NRAS Q61K | |  | 4 | 3 |
| NRAS Q61L | |  | 2 | 1 |
| NRAS Q61R | |  | 2 | 1 |
| NRAS G12C | |  | 1 | 1 |
| NRAS G12D | |  | 1 | 1 |
| NRAS G13R | |  | 1 | 1 |
| Not available^a^ | |  | 16 | 11 |
| **Mutation analysis from BEAMing plasma (*N*= 146)** | | | | |
|  |  |  | **Number** | **Percentage** |
| ***RAS*** |  |  |  |  |
| Mutant |  |  | 57 | 39 |
| WT |  |  | 89 | 61 |
| **Mutation Type** | |  |  |  |
| KRAS G12 | |  | 25 | 17 |
| KRAS G13 | |  | 9 | 6 |
| KRAS A146 | |  | 6 | 4 |
| KRAS Q61 | |  | 4 | 3 |
| KRAS K117 | |  | 1 | 1 |
| KRAS G12/Q61 | |  | 1 | 1 |
| NRAS Q61 | |  | 8 | 5 |
| NRAS G12 | |  | 3 | 2 |

^a^11% considering 146 samples
