# Supplementary figures and images for: Concordance of blood- and tumor-based detection of RAS mutations to guide anti-EGFR therapy in metastatic colorectal cancer
Source: Ann Oncol. 2017 Mar 20;28(6):1294–301. doi: 10.1093/annonc/mdx112 (PMC5834108; doi:10.1093/annonc/mdx112)

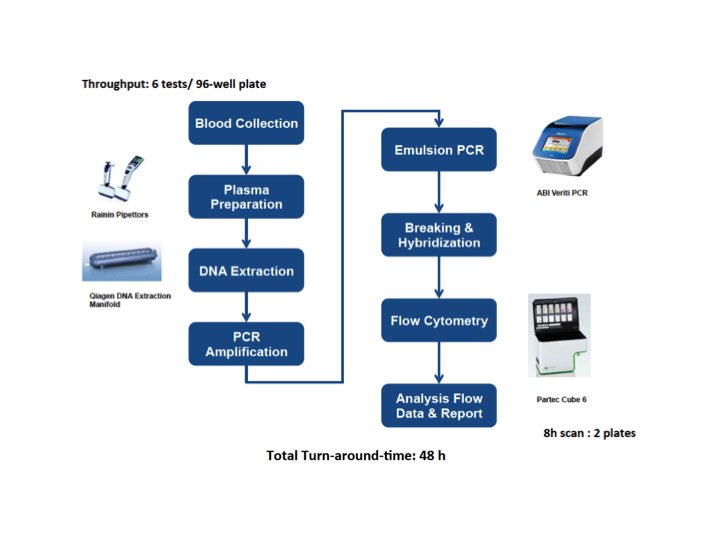

Supplement: mdx112_supp [file mdx112_supp.zip › Supplementary Figure S1 .tif]

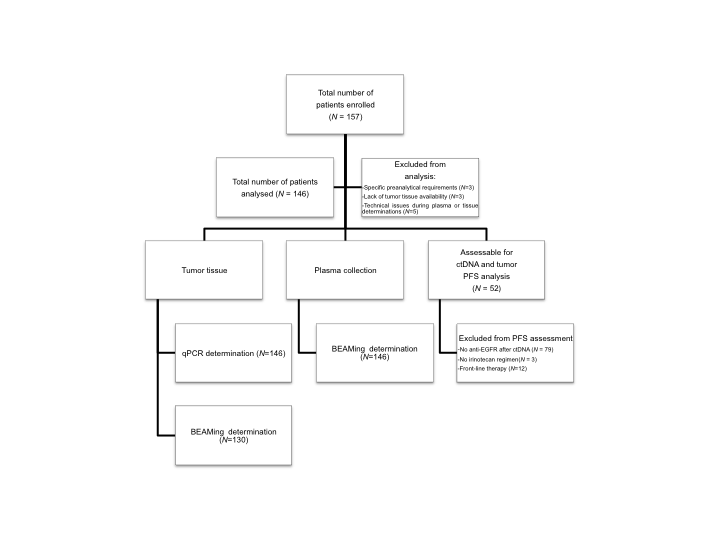

Supplement: mdx112_supp [file mdx112_supp.zip › Supplementary Figure S2 .tif]
